# On the Bioconvective Aspect of Viscoelastic Micropolar Nanofluid Referring to Variable Thermal Conductivity and Thermo-Diffusion Characteristics

**DOI:** 10.3390/bioengineering10010073

**Published:** 2023-01-05

**Authors:** Omar T. Bafakeeh, Kamel Al-Khaled, Sami Ullah Khan, Aamar Abbasi, Charankumar Ganteda, M. Ijaz Khan, Kamel Guedri, Sayed M. Eldin

**Affiliations:** 1 Department of Industrial Engineering, Jazan University, Jazan 82822, Saudi Arabia; 2 Department of Mathematics & Statistics, Jordan University of Science and Technology, P.O. Box 3030, Irbid 22110, Jordan; 3 Department of Mathematics, COMSATS University Islamabad, Sahiwal 57000, Pakistan; 4 Department of Mathematics, University of Azad Jammu and Kashmir, Muzaffarabad 13100, Pakistan; 5 Department of Engineering Mathematics, College of Engineering, Koneru Lakshmaiah Education Foundation, Vaddeswaram 522302, Andhra Pradesh, India; 6 Department of Mathematics and Statistics, Riphah International University I-14, Islamabad 44000, Pakistan; 7 Department of Mechanical Engineering, Lebanese American University, Beirut 1102 2801, Lebanon; 8 Mechanical Engineering Department, College of Engineering and Islamic Architecture, Umm Al-Qura University, P.O. Box 5555, Makkah 21955, Saudi Arabia; 9 Center of Research, Faculty of Engineering, Future University in Egypt, New Cairo 11835, Egypt

**Keywords:** bioconvection flow, viscoelastic micropolar fluid, heat transfer, double diffusion, variable thermal conductivity

## Abstract

The bioconvective flow of non-Newtonian fluid induced by a stretched surface under the aspects of combined magnetic and porous medium effects is the main focus of the current investigation. Unlike traditional aspects, here the viscoelastic behavior has been examined by a combination of both micropolar and second grade fluid. Further thermophoresis, Brownian motion and thermodiffusion aspects, along with variable thermal conductivity, have also been utilized for the boundary process. The solution of the nonlinear fundamental flow problem is figured out via convergent approach via Mathematica software. It is noted that this flow model is based on theoretical flow assumptions instead of any experimental data. The efficiency of the simulated solution has been determined by comparing with previously reported results. The engineering parameters’ effects are computationally evaluated for some definite range.

## 1. Introduction

At present, noteworthy attention is being paid to the study of nanofluids, due to their advanced warm transportation features. Experimental-based investigations report that the thermal performances of nanoparticles may intestinally affect the shape and size of particles, the volume fraction of nanoparticles, the material of particles and traditional base liquids. Several prospective applications of nanofluids related to chemical, biological, mechanical and engineering areas may include cooling of laptop processors, fuel chambers, enhancement of chemical reactions, air purifiers, fission reactions, turbine engines, diagnosis and treatment of tumors, damaging risky tissues in the human body and improving thermal efficiency of raw petroleum materials. Such nanoparticles, originally proposed by Choi, are in the micro-size range (1–100 nm) [1]. Based on the excrementally based observations of [1], nanoparticles have been recommended as the most convenient, cheap and environmentally friendly source for improving thermal conductivity, as comparison to conventional base liquids. Due to such illustrious features, a variety of contributions have been listed by numerous researchers. Buongiorno [2] advised on the most interesting slip features and thermo-physical aspects of nanoparticles, namely thermophoresis and Brownian motion, by developing a non-homogeneous model for the transportation equations. Reddy et al. [3] constituted the slip effects in an unsteady MHD flow of nanofluid along with thermophoresis and Brownian movement features endorsed by a slendering surface. Usman et al. [4] derived a numerical solution for the movement of Casson fluid immersed in nanoparticles with slip effects. The desired flow was objected over a cylinder and the treated numerical method was a collocation technique. Another attempt, focusing on the transportation of disk flow of nanomaterial was predicted by Yin et al. [5]. Madhu et al. [6] performed a theoretical simulation for unsteady Maxwell nanofluid flow via a stretched surface. Turkyilmazoglu [7] initiated the transportation mechanism nanofluid extrusion for free as well as circular jets, and governed equations were treated exactly. We acknowledge another continuation of Turkyilmazoglu [8] regarding the channel flow of nanofluid by using the famous Buongiorno model. Interestingly, the results were also predicted for the pure fluid case and results were reported via the integration technique. Abbasi et al. [9] visualized the Hall mechanism of nanofluid with peristaltic movement under the object of entropy generation. The microorganisms of nanofluid with behavior of slip effects were depicted by Waqas et al. [10]. Some more research on this topic can be seen in [11,12,13,14,15].

It is commonly noted that various transportation systems involve simultaneous heat and mass transfer effects in a moving fluid. In such circumstances, the flux is intricate with respect to both temperature and concentration gradients and notifies as thermodiffusion effects. Such effects may attain prime importance in cases where flow regime governs the different densities. The double diffusion convection phenomenon has key applications in the evaluation of systems which happen with various densities. Agarwal et al. [15] performed investigations for diffusion convective flow of nanofluid in a rotating frame. Raghunatha et al. [16] proved the thermal stability of viscoelastic material for triple diffusion phenomena. Daba et al. [17] discussed the injection onset for tripe diffusive flow in vertical surfaces. Gireesha et al. [18] observed the 3D analysis for tripe diffusion flow numerically. The numerical approach for Prandtl nanofluid flow under double diffusion phenomena was justified by Bilal et al. [19]. Hayat et al. [20] used Cattaneo–Christov relation for double diffusion phenomena with Jeffrey material. Irfan et al. [21] used the variable thermal impact for double diffusion flow. The Soret effects for Maxwell material in a bidirectional regime was investigated by Prasannakumara et al. [22].

The analogous characteristics of non-Newtonian fluids have intrigued engineers and scientist recently owing to their peculiar rheological properties. Various industrial and biological applications have been found for non-Newtonian fluid paints, petroleum products, fiber coating, food industries, engine oils, glass blowing, blood, pharmaceuticals, etc. Due to the diverse features of nonlinear liquids, research is undertaken by scientists in different manners and indicates different mathematical relations for each non-Newtonian model. Listing such rheological models, micropolar fluid is one which explains the behavior of materials under the assessment of a micro-level approach and on a micro basis. The justifications of mathematical expressions for linear as well as couple models can be retained as a limiting case. The basic concept of micropolar fluid was deduced by Eringen [23,24,25,26] and later on many successful contributions were carried out by numerous scientists. For instance, Kabeir [27] examined the stagnation point flow of a micropolar fluid model under the action of a magnetic field. Nazeer et al. [27] and Abbasi et al. [28] worked on heat transport in fluid flow over a diverse geometries. Turkyilmazoglu [29] established some interesting exact solutions for micropolar fluid induced by a flat plate. Sui et al. [30] utilized nonlinear diffusive flow of micropolar fluid over a stretched surface. Sajid et al. [31] numerically analyzed the stagnation point flow over a spiraling disk.

Owing to the above-mentioned chemical, mechanical and engineering significance, the aim here is to present a study of double diffusion flow of viscoelastic micropolar fluid by appending magnetized nanoparticles containing the microorganisms. The viscoelastic micropolar fluid refers to the decomposition of second grade fluid and micropolar fluid refers to the generalized viscoelastic model. The present analysis extends the work of [6] by considering the bioconvection phenomenon, combining magnetic and porous medium effects, interacting the nanoparticles and interpolating double diffusion effects. The analytical expressions which govern the solution of the modeled problem are conveyed through the most pragmatic convergent method, namely the homotopy analysis technique. The realistic physical description and significance are evaluated for each physical parameter. These results can be attributed to the enhancement of thermal efficiency in engineering devices. Further, this model enables us to present simultaneous results for viscous, viscoelastic, micropolar and couple stress fluid.

## 2. Flow Problem

Current continuation reports the bioconvective determination of viscoelastic micropolar nanofluid under the influence of a magnetic field and porous medium. The flow model is based on theoretical flow assumptions. The Buongiorno model is incorporated to see the significance of Brownian motion and thermophoresis impact. We originate a coordinate system from the edge of a stretched surface where velocity component *u* is considered in the *x*-direction while the *v* component is utilized in the *y*-direction. The magnetic field effects are targeted vertically. Further, the temperature, solutal nanofluid concentration and volume fraction are denoted by (T, C, φ). The ambient fluid is retracted by velocity u=bx, where b is constant. Keeping these assumptions in mind, the associated model is explicated as [27,32]:

(1) $$\frac{\partial u}{\partial x}+\frac{\partial v}{\partial y}=0,$$

(2) $$u\frac{\partial u}{\partial x}+v\frac{\partial u}{\partial y}=\frac{k_{1}}{\rho_{f}}\frac{\partial N}{\partial y}+\left(\nu +\frac{k_{1}}{\rho_{f}}\right)\frac{\partial^{2}u}{\partial y^{2}}-k_{0}\left[u\frac{\partial^{3}u}{\partial x\partial y^{2}}+\frac{\partial u}{\partial x}\frac{\partial^{2}u}{\partial y^{2}}-\frac{\partial u}{\partial y}\frac{\partial^{2}u}{\partial x\partial y^{2}}+v\frac{\partial^{3}u}{\partial x^{3}}\right]-\left(\frac{\sigma_{e}B_{0}^{2}}{\rho_{f}}+\frac{\nu \vartheta }{k^{*}}\right)u,$$

(3) $$u\frac{\partial N}{\partial x}+v\frac{\partial N}{\partial y}=\frac{\gamma }{\rho_{f}j}\frac{\partial^{2}N}{\partial y^{2}}-\frac{k_{1}}{\rho_{f}j}\left(2N+\frac{\partial u}{\partial y}\right),$$

(4) $$\begin{array}{l} u\frac{\partial T}{\partial x}+v\frac{\partial T}{\partial y}=\frac{1}{{\left(\rho c_{p}\right)}_{f}}\frac{\partial }{\partial y}\left(K\left(T\right)\frac{\partial T}{\partial y}\right)+\tau_{T}\left[D_{T}\frac{\partial \phi }{\partial y}\frac{\partial T}{\partial y}+\frac{D_{T}}{T_{\infty }}{\left(\frac{\partial T}{\partial y}\right)}^{2}\right]\\ +DK_{TC}\frac{\partial^{2}C}{\partial y^{2}}, \end{array}$$

(5) $$u\frac{\partial C}{\partial x}+v\frac{\partial C}{\partial y}=D_{s}\frac{\partial^{2}C}{\partial y^{2}}+DK_{CT}\frac{\partial^{2}T}{\partial y^{2}},$$

(6) $$u\frac{\partial \varphi }{\partial x}+v\frac{\partial \varphi }{\partial y}=D_{B}\frac{\partial^{2}\varphi }{\partial y^{2}}+\frac{D_{T}}{T_{\infty }}\frac{\partial^{2}T}{\partial y^{2}},$$

(7) $$u\frac{\partial n}{\partial x}+v\frac{\partial n}{\partial y}+\frac{bW_{c}}{\left(C_{w}-C_{\infty }\right)}\left[\frac{\partial }{\partial y}\left(n\frac{\partial C}{\partial y}\right)\right]=D_{m}\left(\frac{\partial^{2}n}{\partial y^{2}}\right),$$

Here $k_{1}$ determines the fluid constant, $k_{0}$ denotes the viscoelastic parameter, $\sigma_{e}$ signifies the electrical conductivity, ϑ stands for the permeability of porous medium, γ spin gradient viscosity, $\rho_{f}$ fluid density, *j* symbolizes the microinertia per unit mass, *N* relates the micro-rotation, $\alpha_{1}$ stands for thermal diffusivity, $D_{B}$ denotes the Brownian diffusion coefficients, $DK_{TC}$ is Dufour diffusivity, $D_{s}$ determine solutal diffusivity, $D_{T}$ notify the thermophoretic diffusion coefficient, $DK_{CT}$ denote Soret diffusivity, $\tau_{T}={\left(\rho c\right)}_{p}/{\left(\rho c\right)}_{f}$ highlights the heat capacity of nanoparticles to heat capacity of fluid ratio.

Current analysis has been performed by articulating the following boundary conditions

(8) $$u=u_{\omega }=bx,\;\;\;v=0,\;\;\;N=0,\;\;\;T=T_{w},\;\;\;C=C_{w},\varphi =\varphi_{w},n=n_{w}\;\;\mathrm{at}\;\;\;y=0,$$

(9) $$u\to 0,\;\frac{\partial u}{\partial y}\to 0,\;u\to 0,\;\;N\to 0,\;T\to T_{\infty },\;\;\;\;C\to C_{\infty },\varphi \to \varphi_{\infty },n\to n_{\infty }\;\mathrm{at}\;\;y\to \infty .$$

For variable conductivity, Equation (4) may be amended by introducing

(10) $$K\left(T\right)=K_{\infty }\left[1+\varepsilon \frac{\left(T-T_{\infty }\right)}{\Delta T}\right],$$

with $K_{\infty }$ (material conductivity) and ε (thermal dependence conductivity factor).

Let us insinuate the appropriate variables to attain the non-dimensional forms [33]

(11) $$u=bxf^{\prime }\left(\eta \right),\;\;v=-\sqrt{bx}f\left(\eta \right),\;N=\sqrt{\frac{b}{\nu }}bxg\left(\eta \right),\;\;\;\eta =\sqrt{\frac{a}{\nu }}y,$$

(12) $$\theta \left(\eta \right)=\frac{T-T_{\infty }}{T_{w}-T_{\infty }},s\left(\eta \right)=\frac{C-C_{\infty }}{C_{w}-C_{\infty }},\phi \left(\eta \right)=\frac{\varphi -\varphi_{\infty }}{\varphi_{w}-\varphi_{\infty }},\chi \left(\eta \right)=\frac{n-n_{\infty }}{n_{w}-n_{\infty }}.$$

After invoking above quantities, Equations (3)–(5) are re-established into following forms

(13) $$\left(1+K\right)f^{\prime \prime \prime }+Kg^{\prime }+{\mathrm{ff}}^{\prime \prime }-{\left(f^{\prime }\right)}^{2}-Haf^{\prime }-k\left[2f^{\prime }f^{\prime \prime \prime }-{\left(f^{\prime \prime }\right)}^{2}-{\mathrm{ff}}^{⁗}\right]=0,$$

(14) $$\left(1+\frac{K}{2}\right)g^{\prime \prime }-K\left(2g+f^{\prime \prime }\right)-f^{\prime }g+g^{\prime }f=0,$$

(15) $$\left(1+\varepsilon \theta \right)\theta^{\prime \prime }+\varepsilon {\left(\theta^{\prime }\right)}^{2}+\mathrm{Pr}\left[f\phi^{\prime }+Nb\theta^{\prime }\phi^{\prime }+Nt{\left(\theta^{\prime }\right)}^{2}+\left(Nd\right)s^{\prime \prime }\right]=0,$$

(16) $$s^{\prime \prime }+Le\left(f\phi^{\prime }\right)+Ld\theta_{yy}=0,$$

(17) $$\phi^{\prime \prime }+Ln\left(f\phi^{\prime }\right)+\frac{Nt}{Nb}\theta^{\prime \prime }=0,$$

(18) $$\chi^{\prime \prime }+Lbf\chi^{\prime }-Pe\left(\phi^{\prime \prime }\left(\chi +\delta_{1}\right)+\chi^{\prime }\phi^{\prime }\right)=0.$$

Governing dimensionless boundary conditions are

(19) $$f^{\prime }\left(0\right)=1,\;\;f\left(0\right)=0,g\left(0\right)=0,\;\theta (0)=1,\;s(0)=1,\phi (0)=1,\chi \left(0\right)=1,$$

(20) $$f^{\prime }\left(\infty \right)\to 0,f^{\prime \prime }\left(\infty \right)\to 0,\;g\left(\infty \right)\to 0,\theta (\infty )\to 0,s(\infty )\to 0,\phi (\infty )\to 0,\chi (\infty )\to 0.$$

We define the most important engineering parameters, such as viscoelastic parameter k, combined parameter $H_{a}$, vortex viscosity parameter K, Prandtl number Pr,, thermophoresis parameter Nt, Brownian motion parameter Nb, modified Dufour number Nd, regular Lewis number Le, Dufour Lewis number Ld, Peclet number Pe, bioconvection Lewis Lb, microorganism concentration difference $\delta_{1}$ and nano Lewis number Ln as follows

$$\begin{array}{c} k=bk_{0}/\nu \rho_{f},\\ Ha=\sqrt{\sigma_{e}B_{0}^{2}/\rho_{f}b}+\nu \vartheta /bk^{*},\\ K=k_{1}/\rho_{f}\nu ,\\ \mathrm{Pr}=\nu /\alpha_{f},\\ N_{t}={\left(\rho c\right)}_{p}D_{T}\left(T_{w}-T_{\infty }\right)/{\left(\rho c\right)}_{f}T_{\infty }\nu ,N_{b}={\left(\rho c\right)}_{p}D_{B}\left(C_{w}-C_{\infty }\right)/{\left(\rho c\right)}_{f}\nu ,\\ Sc=\nu /D_{m},\\ Le=\nu /D_{s},\\ Ld=D_{CT}\left(T_{w}-T_{\infty }\right)/\alpha_{m}\left(C_{w}-C_{\infty }\right),Lb=\frac{\nu }{D_{m}},Pe=\frac{bw_{c}}{D_{m}},Ln=\nu /D_{B},\delta_{1}=\frac{n_{\infty }}{n_{w}-n_{\infty }},\\ Nd=D_{TC}\left(C_{w}-C_{\infty }\right)/\alpha_{m}\left(T_{w}-T_{\infty }\right). \end{array}$$

To illustrates the physical features of shear stress, one must determine the skin friction coefficient in following, which is of flowing form

(21) Cf=τwρuw2,τw=[(μ+k1)∂u∂y+k1N]y=0,CfRex=(1+K)f″(0).

Moreover, the relation for wall couple stress is actuated as

(22) Mx=γ(∂N∂y)y=0ρb2x3,MxRex=KGg′(0).

The definitions of the dimensionless local Nusselt number, local Sherwood number and local nano Sherwood number results in the following expressions

(23) NuxRex−1/2=−θ′(0),   ShxRex−1/2=−s′(0),   ShnRex−1/2=−ϕ′(0),Rex−1/2Nn=−χ′(0).

where Rex=uwx¯/ν is mentioned for the local Reynolds number.

## 3. Homotopy Analysis Method

The nonlinear problems play essentially decisive roles in various disciplines of science, applied engineering and mathematics. Such exciting nonlinearity compels scientists to compose analytical or numerical approaches. Taking into account such analytical methods, the homotopy analysis method (HAM) technique is noted as the preferred dynamical technique due to its well confined accuracy. Unlike the perturbation method, no restriction is noticed for small or larger flow factors. This renewed technique was formerly advocated by Liao [34], and subsequently many equations based on mathematical and interdisciplinary sciences have been successfully treated by introducing this method. Since this technique is familiar from recent years, the details of this method have been omitted here. Readers are referred to some continuations in the literature [33,35,36,37].

## 4. Convergent Region and Solution Confirmations

**Figure 1:**
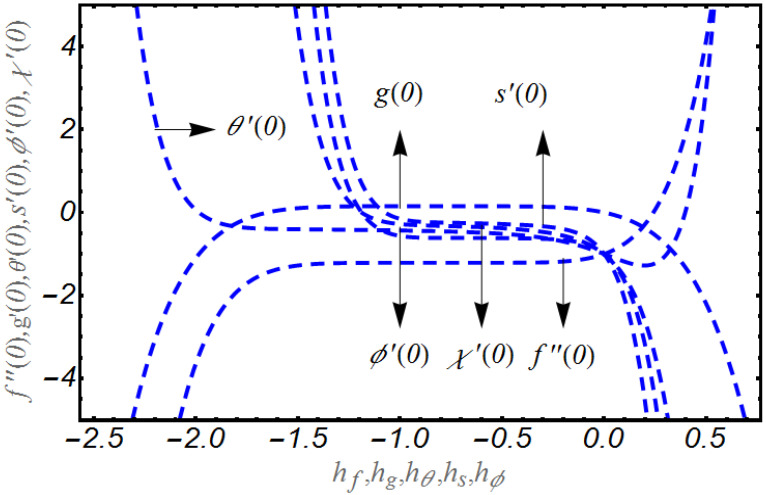
Curves for $f^{\prime \prime }\left(0\right),$ $g^{\prime }\left(0\right),$ $\theta^{\prime }\left(0\right),$ $s^{\prime }\left(0\right)$, $\phi^{\prime }\left(0\right)$ and $\chi^{\prime }\left(0\right)$.

The classical homotopic solution contains some interesting aspects, namely $h_{f},$ $h_{g},$ $h_{\theta },$ $h_{\phi }$ and $h_{s}$ in which are confined some specified regions, to examine the convergence of the HAM method. Obviously, apposite selection of these parameters conferred accurate convergence path and can be determined by h − curves. Figure 1 utilizes such h − curves for $f^{\prime \prime }\left(0\right),$ $g^{\prime }\left(0\right),$ $\theta^{\prime }\left(0\right),$ $s^{\prime }\left(0\right)$ and $\phi^{\prime }\left(0\right),\chi^{\prime }\left(0\right).$ Based on observations, the convergence regions for $f^{\prime \prime }\left(0\right),$ $g^{\prime }\left(0\right),$ $\theta^{\prime }\left(0\right),$ $s^{\prime }\left(0\right)$, $\phi^{\prime }\left(0\right)$ and $\chi^{\prime }\left(0\right)$ have been listed in Table 1.

## 5. Verification of Results

The results are validated in Table 2 for the limiting case with the investigation of El-Kabeir [38]. The claimed results show fine accuracy with these available numerical results.

## 6. Analysis of Results

In this section, graphical results for the involved parameters are presented for k=0.0 and k=0.5.

It is mentioned that in order to vary the flow, parameters have been assigned constant values like K=0.5, Ha=0.2, ε=0.5, Nd=0.2, Pr=0.7,Lb=0.3,Pe=0.2, Le=0.3, Ld=0.3 and Ln=0.3.

**Figure 2:**
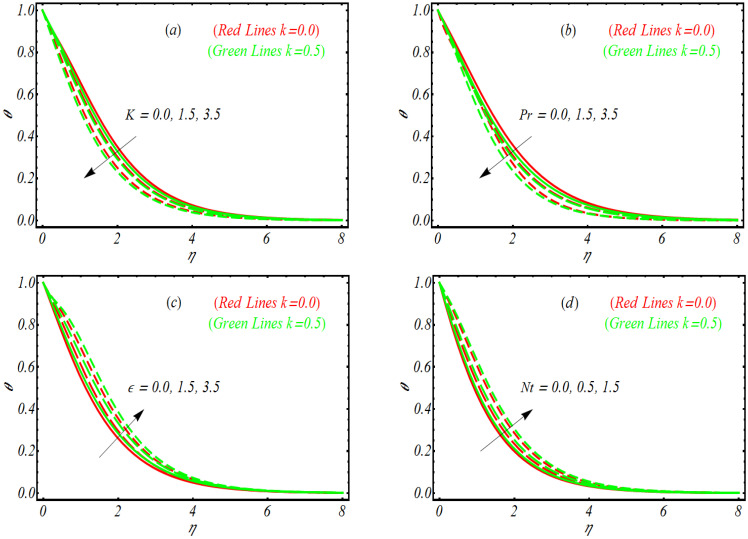

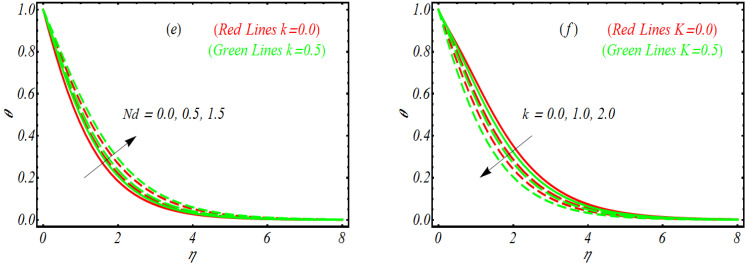
Temperature profile for (**a**) K, (**b**) Pr, (**c**) ε, (**d**) Nt, (**e**) Nd and (**f**) k.

The effects of vortex viscosity factor K, variable thermal conductivity parameter ε, thermophoresis constant Nt, modified Dufour number Nd, Prandtl number Pr, and viscoelastic parameter k on temperature profile θ are visualized by plotting Figure 2a–f. Here, it is remarked that the physical observations for each parameter have been examined for viscoelastic behavior (k=0.5) and without the viscoelastic parameter i.e., k=0.0. First, the variation of K is listed in Figure 2a. The temperature profile decreases by increasing K. In Figure 2b, we conclude that the temperature of nanoparticles for both k=0.0 and k=0.5 declines by varying Pr. This declining trend is relatively dominant for the viscoelastic case k=0.5 as compared to k=0.0. Physically, smaller physical attribution of thermal diffusivity for increasing Pr is noticed, which depressed the temperature. The response of variable thermal conductivity ε on θ is reported in Figure 2c. Following the graphical trend, it is concluded that variable thermal conductivity is more useful to enhance the temperature profile. Further, in contrast to k=0.0, the increasing trend seems to be dominant for k=0.5. From Figure 2d, θ gradually increases when *Nt* is enlarged, that is, due to thermophoresis, the tiny fluid particles shift to the relatively cold region, with a resulting enhancement of temperature. Moreover, the thermal boundary layer is thicker for k=0.5 than k=0.0. The output of θ for modified Dufour number Nd is reported in Figure 2e. The variation of Nd leads to improving the temperature profile and enlarge the thickness of boundary layer. The observations for θ against various values of k are provided in Figure 2f. The reflection of viscoelastic factor reduced thermal flow due to presence of viscosity for both K=0.0 and K=0.5.

**Figure 3:**
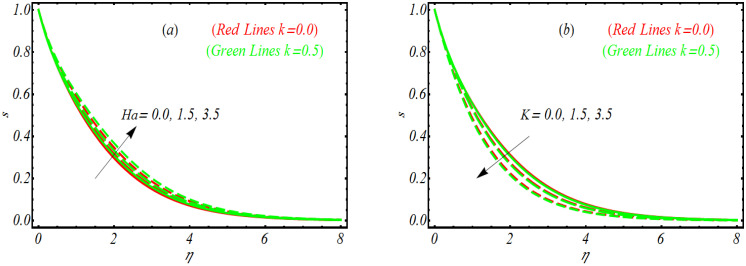

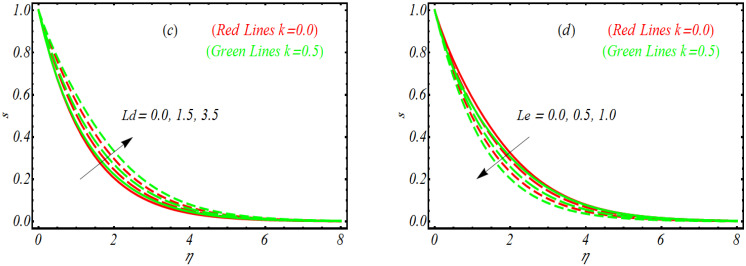
Solutal concentration profile for (**a**) Ha, (**b**) K, (**c**) Ld and (**d**) Le.

In order to look the significance of solutal concentration profile s for combined parameter Ha, vortex viscosity parameter K, Dufour Lewis number Ld and regular Lewis number Le for two different values of k i.e., k=0.0 and k=0.5, Figure 3a–d are suggested. Figure 3a reveals that solutal concentration profile s gets maximum values for Ha for both k=0.0 and k=0.5. Since the combined parameter is a combination of both Hartmann number and porosity parameter. Since Hartmann number has primary relation with Lorentz force which enables the considered profile to enhance. However, the solutal concentration layer is relatively thicker in absence of viscoelastic parameter k=0.0. The influence of K on s has been nominated in Figure 3b. Here a decreasing profile of s has been noted as K varies. Figure 3c reveals that solutal concentration profile s reached at peak as Ld increase. From Figure 3d, it is noted that s increases by increasing Le. Such a trend can be justified physically as the Lewis factor reversely reflects with mass diffusivity. The diffusion rate becomes poor for enlarged values of Le which leads to the decrement of the resulting solutal concentration.

**Figure 4:**
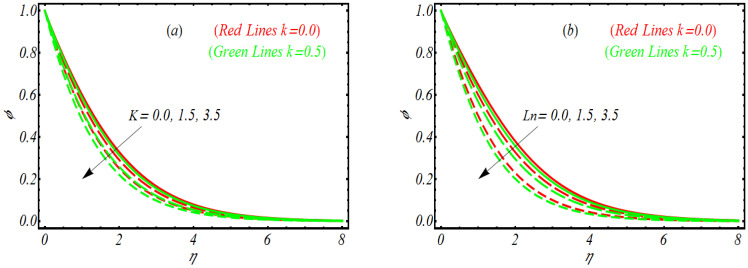

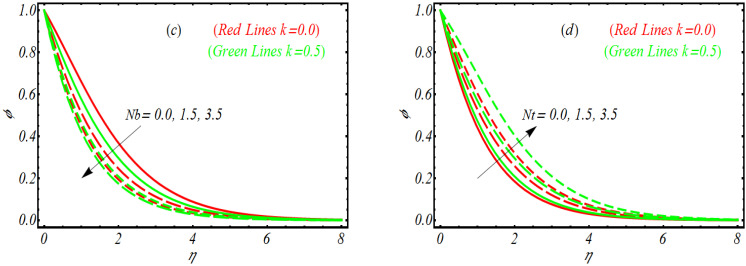
Concentration profile for (**a**) K, (**b**) Ln, (**c**) Nb and (**d**) Nt.

**Figure 5:**
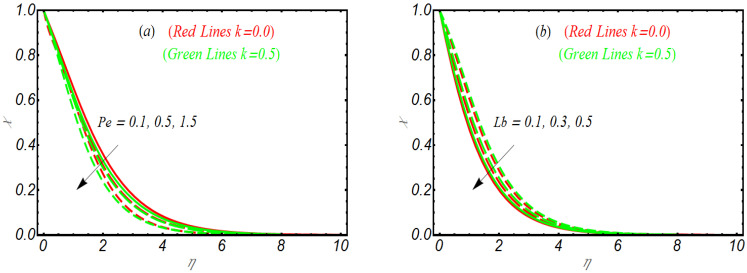
Microorganism profile for (**a**) Pe, (**b**) Lb.

To examine how various involved parameters like vortex viscosity parameter K, nano Lewis number Ln, Brownian motion parameter Nb and thermophoresis parameter Nt influences the concentration of nanoparticles ϕ, Figure 4a–d is prepared. First, we look the graphical illustration of K on ϕ. Like the previous profile, the variation in ϕ for various parameters is observed for the highly viscoelastic case k=0.5 and in absence of second grade fluid, k=0.0. Figure 4a reveals that with the increase of K, the concentration profile for both k=0.0 and k=0.5 decrease effectively. Physically higher values of K are concerned with relatively poor viscosity and hence ϕ declined. Similar to the temperature and solutal concentration profile, the concentration profile is also relatively largely reduced for k=0.5. Figure 4b punctuates that ϕ declines as Ln gets varied. The explanation for the profile of ϕ for Nb is notified in Figure 4c. Again, the larger values associated with Nb depressed the concentration of nanoparticles. The dependence of Nt on ϕ is discussed via Figure 4d. It is easily noted that concentration profile, by varying Nt, enhances. However, for k=0.0, the concentration boundary layer is relatively thin as compared to k=0.5. The results of Figure 5a,b pronounces the change of the microprism profile χ for Peclet number Pe and bioconvection Lewis number Lb. Both parameters reduce the microorganism profile.

The numerical data for of $f^{\prime \prime }\left(0\right)$ and $-g^{\prime }\left(0\right)$ for combined parameter Ha and viscoelastic parameter k are reported in Table 3. Here it is observed that both $f^{\prime \prime }\left(0\right)$ and $-g^{\prime }\left(0\right)$ increases for both Ha and k. However, the such demising variation in $-g^{\prime }\left(0\right)$ is slower as compared to $f^{\prime \prime }\left(0\right).$ Table 4 is made to understand the influence of involved parameters like Ha, Pr, Nt, Nb, ε and K on Nusselt physical quantity, Sherwood constant and motile density number when k=0.1. Here an increasing trend for these quantities has been noted for Pr and K while low numerical values are reported for Ha, Nt, Nb and ε. Studies [39,40,41] highlight the physical significance of heat transfer rate and flow mechanisms [42,43] over diverse surfaces.

## 7. Conclusions

This continuation directed an analytical approach to the double diffusion flow of viscoelastic micropolar fluid by utilizing nanoparticles. Thermophoresis and Brownian effects are also elaborated on in the current analysis. Physical phenomena are formulated by using boundary layer approximations and, later on, analytical simulations have been suggested by using HAM. Some further studies on fluid flow are listed in [44,45,46,47,48]. Main results are given as

- The thermal profile decreases by increasing vortex viscosity parameters while the opposite behavior is visualized for the variable thermal conductivity parameter and thermophoresis parameter. Such a variation trend is more dominant for viscoelastic cases.
- The combined parameter and Dufour Lewis number progressively enlarge the heating and solutal concentration phenomena.
- The lower effects for volume fraction of nanofluid due to nano Lewis number are noted.
- The Nusselt quantity and Sherwood number declined for variable thermal conductivity parameters and thermophoresis parameters.
- The observations made here may play vital role for suggesting and improving manufacturing and diffusion processes.

## Figures and Tables

**Table 1:** Numerical evaluation for convergence region for $h_{f}$, $h_{g}$, $h_{\theta }$, $h_{s}$, and $h_{\phi }$.

Approximation Solutions	Auxiliary Parameters	Convergence Region
f	$h_{f}$	$-1.8\leq h_{f}\leq -0.1$
g	$h_{g}$	$-1.8\leq h_{g}\leq 0$
θ	$h_{\theta }$	$-1.2\leq h_{\theta }\leq -0.1$
s	$h_{s}$	$-1.3\leq h_{s}\leq -0.2$
ϕ	$h_{\phi }$	$-1.7\leq h_{\phi }\leq -0.2$
χ	$h_{\chi }$	$-1.5\leq h_{\chi }\leq 0.0$

**Table 2:** Results verification for $f^{\prime \prime }\left(0\right)$ when α=0.

Ha	El-Kabeir [38]	Present Results
0.2	1.24857	1.24856
0.4	1.29537	1.29535
0.6	1.36988	1.36980

**Table 3:** Numerical values of $f^{\prime \prime }\left(0\right)$ and $-g^{\prime }\left(0\right)$ for number Ha and k.

Ha	k	$f^{\prime \prime }\left(0\right)$	$-g^{\prime }\left(0\right)$
0.1 0.3 0.7	0.2	0.763453 0.810542 0.845234	0.0542344 0.0585465 0.0621414
0.2	0.3 0.5 0.7	0.78522 0.823455 0.893443	0.042234 0.051355 0.5579554

**Table 4:** Numerical values of local Nusselt number and local Sherwood number when k=0.5.

Ha	Pr	Nt	Nb	ε	K	$-\theta^{\prime }\left(0\right)$	$-\phi^{\prime }\left(0\right)$	$-\chi^{\prime }\left(0\right)$
0.0 0.5 1.0	0.7	0.3	0.3	0.1	0.1	0.67456 0.63345 0.60945	0.52678 0.48635 0.44743	0.5056578 0.46658 0.43665
0.5	0.2 0.7 1.0					0.433435 0.48467 0.510535	0.464367 0.51046 0.54426	0.46065 0.48456 0.49547
	0.7	0.0 0.3 0.5				0.432345 0.4165 0.3978	0.550768 0.50235 0.46436	0.452075 0.43867 0.40655
		0.3	0.1 0.3 0.6			0.49361 0.45546 0.43436	0.54646 0.58643 0.60266	0.4568 0.49846 0.51754
				0.0 0.3 0.6		0.440241 0.43656 0.40567	0.492345 0.46654 0.43742	0.4345456 0.4054545 0.391123
				0.1	0.0 0.4 0.7	0.4437 0.5159 0.53755	0.50436 0.53576 0.56747	0.50423 0.53366 0.570547

## Data Availability

All the data are available inside the manuscript.

## Nomenclature

u	velocity
b	stretching constant constant
$H_{a}$	combined parameter
k	viscoelastic parameter
Nt	thermophoresis parameter
Nd	modified Dufour number
Pe	Peclet number
$\delta_{1}$	microorganism concentration difference
Ln	nano Lewis number
ε	thermal dependence conductivity factor
$\sigma_{e}$	electrical conductivity
ϑ	stands for permeability of porous medium
$\rho_{f}$	fluid density
*N*	micro-rotation
$D_{B}$	Brownian diffusion coefficients
$D_{s}$	solutal diffusivity
$DK_{CT}$	Soret diffusivity
C	concentration
T	temperature
K	vortex viscosity parameter
Pr	Prandtl number
Nb	Brownian motion parameter
Le	regular Lewis number
Ld	Dufour Lewis number
Lb	bioconvection Lewis
$k_{1}$	fluid constant
$K_{\infty }$	material conductivity
$k_{0}$	viscoelastic parameter
γ	spin gradient viscosity
*j*	microinertia per unit mass
$\alpha_{1}$	thermal diffusivity
$DK_{TC}$	Dufour diffusivity
$D_{T}$	thermophoretic diffusion coefficient
$\tau_{T}={\left(\rho c\right)}_{p}/{\left(\rho c\right)}_{f}$	heat capacity of nanoparticles to heat capacity of fluid ratio.
